# Oral anticoagulant use among Medicare patients newly diagnosed with venous thromboembolism (VTE): Factors associated with treatment status

**DOI:** 10.1371/journal.pone.0321106

**Published:** 2025-04-17

**Authors:** James C. Coons, Aolin Wang, Dominick Latremouille-Viau, Cristina Russ, Dong Cheng, Robert Stellhorn, Feng Dai, David R. Steffen, Abigail Zion, Serina Deeba, Dionne M. Hines

**Affiliations:** 1 Department of Pharmacy, UPMC Presbyterian Hospital, Pittsburgh, Pennsylvania, United States of America; 2 University of Pittsburgh School of Pharmacy, Pittsburgh, Pennsylvania, United States of America; 3 Analysis Group, Inc., New York, New York, United States of America; 4 Analysis Group, Inc., Montreal, Quebec, Canada; 5 Pfizer Inc., New York, New York, United States of America; 6 Bristol Myers Squibb, Lawrenceville, New Jersey, United States of America; 7 Analysis Group, Inc., Boston, Massachusetts, United States of America; Juntendo University: Juntendo Daigaku, JAPAN

## Abstract

**Objective:**

This study aimed to describe oral anticoagulant (OAC) use among patients with venous thromboembolism (VTE).

**Materials and methods:**

This study included Medicare fee-for-service beneficiaries (data from 1/1/2014-12/31/2019) newly diagnosed with VTE. Factors associated with being untreated with OACs in the first month from VTE (vs. OAC-treated), with receiving direct-acting OACs ([DOACs] vs. warfarin), and with extended OAC treatment (>3 months) were assessed using multivariable logistic regressions.

**Results:**

Overall, 169,928 patients with VTE (50.3% OAC-untreated) were included. Among the 49.7% OAC-treated patients, 74.0% used DOACs and 62.5% had extended OAC treatment. Factors associated with being untreated with OACs in the first month from VTE (odds ratio; 95% confidence interval) included Hispanic ethnicity (vs. White;1.35; 1.29–1.42), having part D low-income subsidy (1.14; 1.07, 1.20), and comorbidities such as cardiovascular diseases. Among the OAC-treated cohort, patients with index VTE diagnosis in the emergency room (vs. outpatient) setting had higher odds of receiving DOAC vs. warfarin; patients with pulmonary embolism diagnosis (vs. deep vein thrombosis) had higher odds of extended OAC treatment.

**Conclusions:**

In this study of Medicare patients newly diagnosed with VTE, half of the patients were not treated with OAC in the first month from initial diagnosis. Factors such as Hispanic ethnicity, having low-income subsidy, and comorbidity burden were found to be associated with being untreated with OAC. Among OAC-treated patients, the majority were treated with DOAC vs. warfarin. Interestingly, more than a third of OAC-treated patients were not treated beyond 3 months, which warrants further investigation.

## Introduction

Venous thromboembolism (VTE), comprising both deep vein thrombosis (DVT) and pulmonary embolism (PE), is a recognized major public health problem in the United States (US), causing significant morbidity and mortality with an estimated 1,036,000 persons in the US affected in 2019 [1]. Clinically, patients with VTE may manifest as having DVT, PE, or both. Approximately two-thirds of patients will present with DVT and the remaining one-third present with PE [2].

VTE is a life-threatening condition; 30-day case fatality rates following an acute episode in the range of 10% – 30% have been reported [2–4]. The majority of deaths from VTE are due to PE, which is currently the third leading cause of cardiovascular death in the US [2–5]. The risk of a recurrence of thromboembolism is highest in the days and weeks following an event, thus early anticoagulation treatment is considered critical in preventing recurrence, death, and mitigating longer-term sequelae and complications [6,7].

Current treatment guidelines by the American College of Chest Physicians (ACCP) and by the American Society of Hematology (ASH) recommend an initial course of oral anticoagulants (OACs) for 3–6 months, generally (with some exceptions) favoring use of direct-acting oral anticoagulants (DOACs; apixaban, dabigatran, edoxaban, rivaroxaban) over warfarin in patients with an acute episode of VTE [8,9]. DOACs have thus become the cornerstone for secondary prevention of VTE in recent years [10]. Some exceptions to this are where warfarin may be more appropriate, notably in pregnancy, chronic kidney disease, and antiphospholipid syndrome [11].

Clinical decisions about initiation of OAC treatment and extension of anticoagulation beyond the initial 3-month recommended course for secondary prevention of VTE take into account the benefit of thrombolytic therapy versus risk for bleeding, which can be a life-threatening adverse event [6,12–15]. DOACs have been shown to have significantly lower risk of bleeding compared to warfarin, with comparable or superior efficacy [6,16]. While recently there has been much attention paid to initiatives to improve primary prevention of VTE in view of the improved safety profile of DOACs, especially in hospitalized and higher-risk patients [13,15], the literature on secondary prevention and OACs use for VTE is more scarce.

This study aimed to provide insights into current treatment of patients presenting with DVT and/or PE by describing OAC use among Medicare beneficiaries newly diagnosed with VTE and examining the factors associated with not receiving OAC treatment within 30 days following an initial VTE event. In addition, this study assessed factors associated with treatment with DOACs versus warfarin, and factors associated with extended OAC treatment beyond three months post the initial VTE event.

## Materials and methods

### Data source and sample selection

This retrospective cohort study used data from the CMS Medicare fee-for-service (FFS) claims database. The database includes information on inpatient departments, outpatient departments, carrier claims, prescription drugs, and beneficiaries’ enrollment data. Data were de-identified and comply with the patient requirements of the Health Insurance Portability and Accountability Act (HIPAA) of 1996. An exemption from an institutional review board was obtained per Title 45 of CFR, Part 46.101(b)(4) (https://www.hhs.gov/ohrp/regulations-and-policy/regulations/45-cfr-46/#46.101).

Medicare beneficiaries aged ≥65 years newly diagnosed with VTE (primary or secondary position) in the inpatient or ambulatory (including emergency room [ER]) setting during January 1, 2015–December 31, 2019 were included in this study. Patients whose first VTE claim was in the ambulatory setting were additionally required to have a second VTE claim in the inpatient or ambulatory setting on a separate date within 14 days following the first claim. Additional eligibility criteria included: continuous Medicare Parts A, B, and D enrollment ≥12 months before (baseline period) and ≥30 days after first VTE diagnosis (index VTE); no diagnosis for atrial fibrillation/flutter, mechanical heart valve, inferior vena cava filter, antiphospholipid syndrome, nor pregnancy at any time during the study period; no diagnosis for cancer in the 6 months prior to index VTE through 30 days post index VTE; and, no OAC or prophylactic parenteral anticoagulant treatment in the 12 months prior to the index VTE. The first observed OAC received on or within 30 days after the index VTE date was defined as the index OAC.

The final analytic sample included two patient cohorts defined according to whether or not there was a pharmacy claim for OAC on or within 30 days after the index VTE event (the 30-day window was considered timely treatment given the acute nature of VTE and high risk of recurrence). The cohort without timely treatment (referred to as “untreated cohort”) comprised patients without a claim for an OAC during the 30-day period from index VTE. The cohort with timely treatment (referred to as “OAC-treated cohort”) consisted of patients with at least 1 OAC claim within that same period. The OAC-treated cohort was further classified based on the type of index OAC into: (1) warfarin cohort, comprising patients whose index OAC was warfarin (patients who received bridged therapy with low molecular weight heparin were included), and (2) DOACs cohort, comprising patients whose first OAC received was either apixaban, dabigatran, rivaroxaban, or edoxaban. In addition, among the subgroup of patients in the OAC-treated cohort with continuous health plan enrollment for >5 months post-index VTE, those with continuous use of an index OAC for at least 3 months without a gap of >30 days were included in the extended-treatment cohort.

### Outcomes and statistical analysis

Outcomes assessed included proportion of patients treated with OAC, type of index OAC, and duration of index OAC. Descriptive statistics were used to summarize baseline patient characteristics, as well as OAC treatment use post-VTE event. Continuous variables were described using means and standard deviations (SDs) whereas categorical variables were described using frequencies and proportions. Multivariable logistic regression was used to examine factors associated with: (1) being untreated with OAC versus treated with OAC during the first 30 days from index VTE among patients with VTE overall; (2) receiving DOAC treatment versus warfarin among OAC-treated patients; and (3) receiving extended OAC treatment beyond 3 months versus no extended treatment among OAC-treated patients who had continuous health plan enrollment for >5 months after the index date. Variables were selected based on potential clinical and policy relevance and importance. Subgroup analyses evaluated factors associated with being untreated with OAC by index VTE type (i.e., DVT or PE [with or without DVT]). Sensitivity analyses were carried out to evaluate factors associated with extended treatment beyond 6 months among OAC-treated patients who had continuous health plan enrollment for >8 months after the index date. Results from multivariable logistic regression were reported as odds ratios (ORs) with 95% confidence intervals (CIs). A two-sided p<0.05 was considered statistically significant. All analyses were conducted using SAS version 9.4 (SAS Institute Inc., Cary, North Carolina, USA).

## Results and discussion

### Patient selection and OAC use

**Fig 1:**
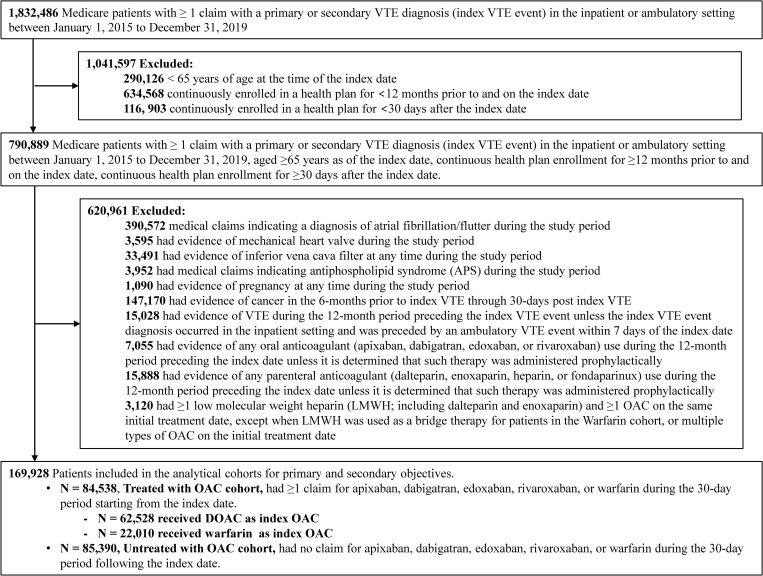
Sample Selection.

A total of 169,928 patients with a VTE diagnosis were included in the analytical cohorts, comprising 84,538 (49.7%) in the OAC-treated cohort and 85,390 (50.3%) in the untreated cohort (Fig 1). Among the OAC-treated patients, 62,528 (74.0%) received DOAC and 22,010 (26.0%) received warfarin as index OAC (Fig 1). Among those patients receiving DOAC, 32,480 (51.9%) were treated with apixaban, 29,324 (46.9%) with rivaroxaban, and the remaining patients were treated with dabigatran (708 [1.1%]) or edoxaban (16 [0.03%]. Due to the small sample size, patients receiving edoxaban were not further evaluated). There were 8,999 patients (5.3%) in the overall VTE cohort who received low molecular weight heparin (LMWH; dalteparin or enoxaparin) not classified as bridge therapy to warfarin (bridge therapy was defined as using LMWH ≤14 days after the index VTE event before using warfarin as their first observed OAC) within the first 30 days of the index VTE event. These patients were not further examined in the analysis.

### Baseline demographic and clinical characteristics

Mean ages (Standard Deviation [SD]) were 76.2 years (SD = 7.6) in the OAC-treated, and 77.3 years (SD = 8.2) in the untreated cohort (Table 1). The majority of patients in both cohorts were female, 62.9% and 66.4%, and non-Hispanic white, 82.6% and 75.4%, in the OAC-treated and untreated cohorts, respectively. Black patients comprised 9.4% and 11.9% of OAC-treated and untreated patients, respectively. The highest regional representation was from the South, with 36.3% of OAC-treated and 39.4% of untreated cohorts. There was a higher percentage of untreated patients compared with OAC-treated patients that were covered by the Medicare Part-D low-income subsidy (31.8% vs. 22.2%, respectively) or with dual eligibility with Medicaid (28.1% vs. 19.2%, respectively). A majority of patients in both treatment cohorts received their index VTE diagnosis in the inpatient setting – 71.4% in the untreated cohort and 63.0% in the OAC-treated cohort.

In both cohorts, more patients had DVT only compared with PE (with or without DVT). In the OAC-treated cohort, 51.8% had DVT only and 48.2% had PE, while 70.3% of the untreated cohort had DVT only and 29.7% had PE (Table 1). Mean Charlson Comorbidity Index (CCI) scores were 2.0 (SD = 2.0) and 3.0 (SD = 2.5) for the OAC-treated and untreated cohorts, respectively. Common comorbidities in both OAC-treated and untreated cohorts were hypertension (77.9% and 84.7%), hyperlipidemia (62.6% and 66.9%), anemia (28.7% and 44.2%), and ischemic heart/coronary artery disease (30.2% and 42.4%), respectively. Over half of patients in both OAC-treated and untreated cohorts used vasodilators (58.7% and 61.1%, respectively), or corticosteroids (56.6% and 52.0%, respectively), during baseline period.

**Table 1:** Baseline^1^ characteristics of patients newly diagnosed with VTE.

	Treated with OAC Cohort (N = 84,538)	Untreated with OAC Cohort^2^ (N = 85,390)
**Demographics at index date** ^**3**^		
**Age (years)**		
Mean ± SD	76.2 ± 7.6	77.3 ± 8.2
Categories, N (%)		
65–74 years	41,227 (48.8%)	37,580 (44.0%)
75–79 years	17,364 (20.5%)	16,599 (19.4%)
≥80 years	25,947 (30.7%)	31,211 (36.6%)
**Sex, N (%)**		
Female	53,176 (62.9%)	56,686 (66.4%)
**Race/ethnicity,**^**4**^ **N (%)**		
American Indian/Alaska Native	341 (0.4%)	417 (0.5%)
Asian/Pacific Islander	1,047 (1.2%)	2,413 (2.8%)
Black	7,981 (9.4%)	10,129 (11.9%)
Hispanic	3,716 (4.4%)	6,618 (7.8%)
Non-Hispanic White	69,826 (82.6%)	64,389 (75.4%)
Other	428 (0.5%)	581 (0.7%)
Unknown	1,199 (1.4%)	843 (1.0%)
**Geographic region, N (%)**		
Northeast	15,619 (18.5%)	17,148 (20.1%)
South	30,679 (36.3%)	33,674 (39.4%)
Midwest	22,549 (26.7%)	19,265 (22.6%)
West	15,530 (18.4%)	15,061 (17.6%)
Other^5^	161 (0.2%)	242 (0.3%)
**Special types of Medicare coverage at index date, N (%)**		
Dual Eligibility^6^	16,196 (19.2%)	23,966 (28.1%)
Low-income subsidy	18,804 (22.2%)	27,138 (31.8%)
**Index year, N (%)**		
2015	14,395 (17.0%)	16,682 (19.5%)
2016	16,011(18.9%)	16,931 (19.8%)
2017	16,698 (19.8%)	18,180 (21.3%)
2018	18,572 (22.0%)	16,905 (19.8%)
2019	18,862 (22.3%)	16,692 (19.5%)
**Setting of index VTE event,**^**7**^ **N (%)**		
Inpatient	53,290 (63.0%)	60,937 (71.4%)
Outpatient only	13,780 (16.3%)	18,208 (21.3%)
ER (without inpatient)	17,468 (20.7%)	6,245 (7.3%)
**Diagnosing physician specialty,**^**8**^ **N (%)**		
Diagnostic radiologist	64,301 (76.1%)	52,214 (61.1%)
Emergency medicine	4,439 (5.3%)	2,349 (2.8%)
Hematologist	607 (0.7%)	715 (0.8%)
Pulmonologist	965 (1.1%)	2,079 (2.4%)
Cardiologist	2,563 (3.0%)	4,722 (5.5%)
Primary care	8,059 (9.5%)	15,007 (17.6%)
Other/unknown	3,604 (4.3%)	8,304 (9.7%)
**Type of VTE, N (%)**		
DVT only	43,799 (51.8%)	60,044 (70.3%)
*Acute thrombosis, proximal DVT, lower extremity or abdominal*	43,342 (51.3%)	55,115 (64.5%)
PE with or without DVT	40,739 (48.2%)	25,346 (29.7%)
**VTE etiology, N (%)**		
Provoked	17,517 (20.7%)	25,104 (29.4%)
Unprovoked	67,021 (79.3%)	60,286 (70.6%)
**Comorbidity profile in the baseline period**		
Charlson Comorbidity Index (CCI) using Quan 2005,^9^ mean ± SD	2.0 ± 2.0	3.0 ± 2.5
**Individual comorbidities, N (%)**		
AIDS	314 (0.4%)	387 (0.5%)
Alcohol abuse	1,866 (2.2%)	3,290 (3.9%)
Anemia	24,299 (28.7%)	37,765 (44.2%)
Central venous catheter	3,053 (3.6%)	8,416 (9.9%)
Cerebrovascular disease	14,597 (17.3%)	24,948 (29.2%)
Hematologic disorders associated with bleeding	4,835 (5.7%)	7,893 (9.2%)
Ischemic heart/coronary artery disease	25,490 (30.2%)	36,229 (42.4%)
Dementia	12,528 (14.8%)	20,561 (24.1%)
Dyspepsia or stomach discomfort	20,919 (24.7%)	25,313 (29.6%)
Hemiplegia or paraplegia	1,623 (1.9%)	4,289 (5.0%)
Hyperlipidemia	52,931 (62.6%)	57,168 (66.9%)
Obesity	23,389 (27.7%)	22,780 (26.7%)
Pneumonia	9,918 (11.7%)	16,542 (19.4%)
Rheumatologic disease	4,452 (5.3%)	4,969 (5.8%)
Sleep apnea	11,539 (13.6%)	10,885 (12.7%)
Spinal cord injury	344 (0.4%)	668 (0.8%)
Thrombophilia	1,338 (1.6%)	1,329 (1.6%)
Varicose veins	4,232 (5.0%)	5,599 (6.6%)
Congestive heart failure	10,858 (12.8%)	20,385 (23.9%)
Diabetes	25,922 (30.7%)	34,251 (40.1%)
Hypertension	65,865 (77.9%)	72,338 (84.7%)
Renal disease	17,210 (20.4%)	23,571 (27.6%)
Liver disease	5,890 (7.0%)	8,267 (9.7%)
COPD	15,687 (18.6%)	23,524 (27.5%)
Peptic ulcer disease	1,922 (2.3%)	3,363 (3.9%)
Inflammatory bowel disease	1,378 (1.6%)	1,518 (1.8%)
Peripheral vascular disease	14,772 (17.5%)	23,866 (27.9%)
Baseline any bleed	16,978 (20.1%)	25,801 (30.2%)
**Recent history of falls, N (%)**	10,555 (12.5%)	15,676 (18.4%)
**Fracture, N (%)**	7,522 (8.9%)	11,624 (13.6%)
**Orthopedic/pelvic surgeries, N (%)**	5,502 (6.5%)	6,704 (7.9%)
**HAS-BLED Score, mean ± SD**	1.5 ± 0.6	1.8 ± 0.7
**Baseline medication use, N (%)**		
Antiarrhythmic	3,810 (4.5%)	3,534 (4.1%)
Statins	41,005 (48.5%)	42,450 (49.7%)
Anti-platelets	6,275 (7.4%)	10,385 (12.2%)
Aromatase inhibitors	79 (0.1%)	54 (0.1%)
Beta blockers	28,273 (33.4%)	32,371 (37.9%)
Gastroprotective agents	29,664 (35.1%)	31,959 (37.4%)
ACE inhibitors	25,112 (29.7%)	25,007 (29.3%)
ARB	18,503 (21.9%)	19,819 (23.2%)
NSAIDs	27,656 (32.7%)	26,325 (30.8%)
Corticosteroids	47,843 (56.6%)	44,429 (52.0%)
Loop diuretics	14,935 (17.7%)	19,046 (22.3%)
Potassium-sparing diuretics	2,761 (3.3%)	3,228 (3.8%)
Thiazide diuretics	10,991 (13.0%)	10,658 (12.5%)
Vasodilators	49,599 (58.7%)	52,135 (61.1%)
PPIs	26,021 (30.8%)	27,510 (32.2%)
SSRIs	16,562 (19.6%)	18,146 (21.3%)

Abbreviations: ACE = angiotensin-converting enzyme; AIDS = acquired immune deficiency syndrome; ARB = angiotensin receptor blockers; COPD = chronic obstructive pulmonary disease; DVT = deep vein thrombosis; ER = emergency room; HAS-BLED = Hypertension, Abnormal liver/renal function, Stroke history, Bleeding history or predisposition, Labile INR, Elderly, Drug/alcohol usage; LMWH = low molecular weight heparin; NSAID = non-steroidal anti-inflammatory drug; OAC = oral anticoagulant; PE = pulmonary embolism; PPI = proton pump inhibitors; SSRI = selective serotonin reuptake inhibitors; VTE = venous thromboembolism.

Notes:

^1^The baseline period is defined as the 12 months prior to index date.

^2^Includes patients without any OAC treatment during the study period, patients with OAC treatment started after 30 days, and patients with LMWH but no OAC.

^3^The index date is defined as the start of the study period, January 1, 2014.

^4^Race/ethnicity are presented as mutually exclusive categories.

^5^U.S. territories such as Puerto Rico and Virgin Island.

^6^This category includes patients enrolled in Medicare and getting full Medicaid benefits (i.e., enrolled in Medicaid) and/or assistance with Medicare premiums or cost-sharing through the Medicare Savings Program.

^7^Patients with claims from multiple settings on their index date were classified as having their index VTE event in the setting that is the first in the following order: inpatient, ER, outpatient. Accordingly, the settings are mutually exclusive.

^8^Patients who saw several types of physicians on their index date were classified as having seen the physician with the first specialty in the following order: diagnostic radiologist, emergency medicine, hematologist, pulmonologist, cardiologist, primary care, or other/unknown.

^9^The Charlson Comorbidity Index was defined based on criteria by Charlson (1987), adapted by Deyo (1992), and updated by Quan (2005). Citation: Quan, H. et al., 2005. Coding algorithms and for defining comorbidities in ICD-9-CM and ICD-10 administrative data. Medical Care, 43(11),pp.1130–1139.

### Predictors of OAC treatment pattern

**Fig 2:**
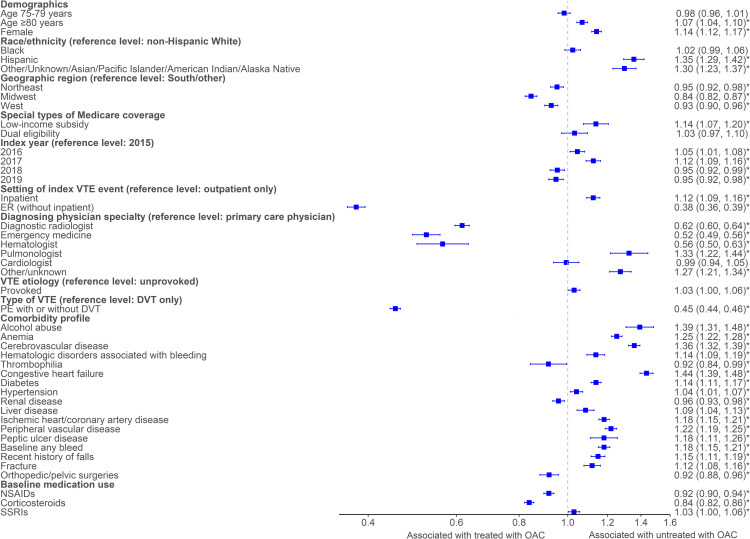
Factors associated with being untreated vs. treated with OAC.^*^P-value < 0.05.

Results from the multivariable logistic regression model revealed several sociodemographic and clinical factors that were related to being untreated within the first 30 days following the VTE event. For example, compared with non-Hispanic White patients, Hispanic patients and those classified as “Other” had 1.35 (OR [95% CI]: 1.35 [1.29, 1.42]) and 1.30 (OR: 1.30 [1.23, 1.37]) times the odds of being untreated, respectively. Patients eligible for Medicare Part-D low-income coverage had 14% higher odds of being untreated than those ineligible for the subsidy (OR: 1.14 [1.07, 1.20]). The five most impactful baseline comorbidities that were associated with increased odds of being untreated were: congestive heart failure (OR: 1.44 [1.39, 1.48]); alcohol abuse (OR: 1.39 [1.31, 1.48]); cerebrovascular disease (OR: 1.36 [1.32, 1.39]); anemia (OR: 1.25 [1.22, 1.28]); and peripheral vascular disease (OR: 1.22 [1.19, 1.25]). Setting of index VTE diagnosis was identified as a factor influencing treatment, with 62% decreased odds of being untreated observed among those with their index VTE event in an ER (without hospitalization) compared to outpatient (OR: 0.38 [0.36, 0.39]). Conversely, having the index event occur in the inpatient setting (compared to outpatient) increased odds of being untreated (OR: 1.12 [1.09, 1.16]). Fig 2 displays all factors significantly associated with being untreated.

Having VTE diagnosed by a specialist (compared to primary care physician) was associated with decreased odds of being untreated. Patients diagnosed by a diagnostic radiologist had 38% lower odds of being untreated (OR: 0.62 [0.60, 0.64]), and odds of being untreated were 48% lower odds if diagnosed by an emergency medicine specialist (OR: 0.52 [0.49, 0.56]), and 44% lower if diagnosed by a hematologist (OR: 0.56 [0.50, 0.63]). Having a PE as the index VTE type was associated with 55% lower odds of being untreated with OAC (OR: 0.45 [0.44, 0.46]).

A sensitivity analysis was performed that included medications used to treat key morbidities rather than underlying comorbidities as covariates, and results were largely and qualitatively consistent with the primary analysis (S1 Fig).

### Subgroup analyses by VTE type

Of the 169,928 patients in the overall VTE cohort, 103,843 (61.1%) had DVT only and 66,085 (38.9%) had PE (with or without DVT) as index VTE type (S1 Table). There were 60,044 (57.8%) untreated patients among patients with DVT whereas 25,346 (38.4%) patients with PE were untreated (S1 Table).

Baseline characteristics were largely similarly distributed between the two VTE types and with respect to OAC-treated vs. untreated cohorts when compared with the overall sample, with some exceptions (S1 Table). Among patients with DVT only, 17,776 (40.6%) of the OAC-treated and 38,569 (64.2%) of the untreated cohort had the index VTE event in the inpatient setting. Among patients with PE as the index VTE event, higher proportions of patients had the event in the inpatient setting compared with DVT only, and these were relatively similarly distributed in the OAC-treated [35,514 (87.2%)] and untreated [22,368 (88.3%)] cohorts. The prevalence of certain individual comorbidities such as ischemic heart/coronary artery disease, COPD, obesity, pneumonia, and sleep apnea appeared to be higher in patients with PE, compared with DVT only, across both OAC-treated and untreated cohorts (S1 Table).

Results from multivariable logistic regression of odds of being untreated according to VTE type (S2 Table) indicated that in patients with DVT, odds of being untreated were higher when the index event occurred in the inpatient setting compared with outpatient only (OR: 1.48 [1.43, 1.53]) and in patients with a baseline comorbidity of having any bleeding versus no bleeding (OR: 1.22 [1.18, 1.26]). Patients with DVT had decreased odds of being untreated in more recent years compared with 2015, by 18% in 2018 (OR: 0.82 [0.78, 0.85]) and by 20% in 2019 (OR: 0.80 [0.77, 0.83]). Conversely, this trend was reversed in patients with PE, with higher odds of being untreated observed in 2016 (OR: 1.50 [1.41, 1.59]), in 2017 (OR: 1.58 [1.49, 1.68]), in 2018 (OR: 1.33 [1.26, 1.41]) as well as in 2019 (OR: 1.36 [1.28, 1.44]). Also, in patients with PE, advanced age (80+ compared with ages 65–74) was associated with increased odds of being untreated (OR: 1.21 [1.16, 1.26]), as was being eligible as a Medicare Part D low-income subsidy (OR: 1.27 [1.16, 1.39]). PE patients with a recent history of falls were also at increased odds of being untreated (OR: 1.28 [1.22, 1.35]), as were those with a fracture (OR: 1.25 [1.17, 1.33]). Compared to PE patients in the South, decreased odds of being untreated was observed in patients in the Midwest (OR: 0.82 [0.78, 0.85]) and West (OR: 0.83 [0.79, 0.87]). Having the index PE event occurring in an inpatient compared with an outpatient setting was associated with decreased odds of being untreated (OR: 0.46 [0.43, 0.50]) (S2 Table).

### Subgroup analysis of OAC treated patients (DOAC or warfarin)

DOAC (vs. warfarin) became increasingly more prevalent as treatment following an index VTE event throughout our indexing period (2015–2019). Among OAC-treated patients, the proportions of patients who initiated DOAC between 2015 and 2019 were 48.6%, 65.3%, 74.8%, 84.8%, and 89.2% for each respective year. The DOAC and warfarin cohorts had similar comorbidity profiles in the baseline period; mean CCI was 2.0 (SD = 2.0) for the DOAC cohort and 2.2 (SD = 2.2) for the warfarin cohort. There were similar proportions of patients in both cohorts with obesity as a baseline comorbidity; 17,570 (28.1%) in the DOAC cohort and 5,819 (26.4%) in the warfarin cohort.

**Fig 3:**
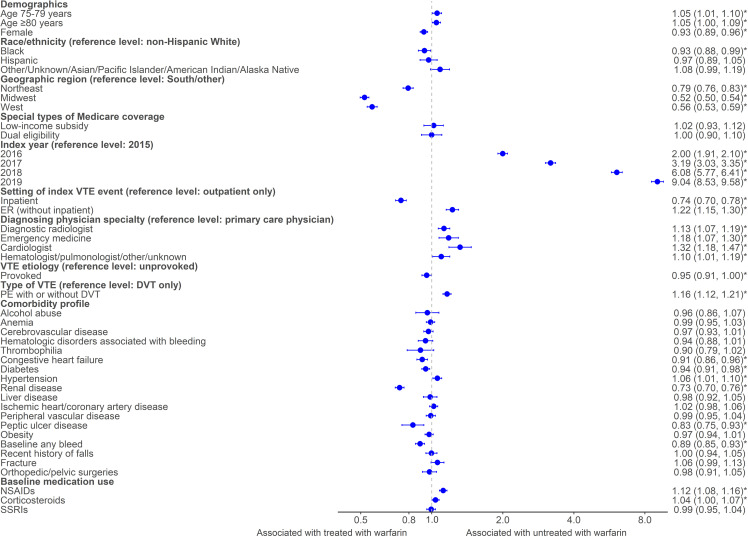
Factors associated with being treated with DOAC vs. warfarin among treated with OAC subgroup. *P-value < 0.05.

Increased odds of being treated with DOAC versus warfarin were noted for several factors (Fig 3). Relative to 2015, odds of DOAC treatment increased two-fold in 2016 (OR: 2.00 [1.91, 2.10]), over three-fold in 2017 (OR: 3.19 [3.03, 3.35]), over six-fold in 2018 (OR: 6.08 [5.77, 6.41]), and over nine-fold in 2019 (OR: 9.04 [8.53, 9.58]). The odds of treatment with DOAC versus warfarin were 22% higher for an index VTE event occurring in the ER relative to the outpatient-only setting (OR: 1.22 [1.15, 1,30]). Conversely, factors that decreased the odds of being treated with DOAC versus warfarin included region (relative to the South): 21% lower odds for those patients in the Northeast (OR: 0.79 [0.76, 0.83]), 48% lower odds for those in the Midwest (OR: 0.52 [0.50, 0.54]), and 44% lower for those in the West (OR: 0.56 [0.53, 0.59]). Having an index VTE in the inpatient setting was associated with 26% lower odds of DOAC treatment (OR: 0.74 [0.70–0.78]), and patients with renal disease as a baseline comorbidity had 27% lower odds of DOAC treatment (OR: 0.73 [0.70, 0.76]).

### Factors associated with extended treatment beyond three months in OAC-treated patients

**Fig 4:**
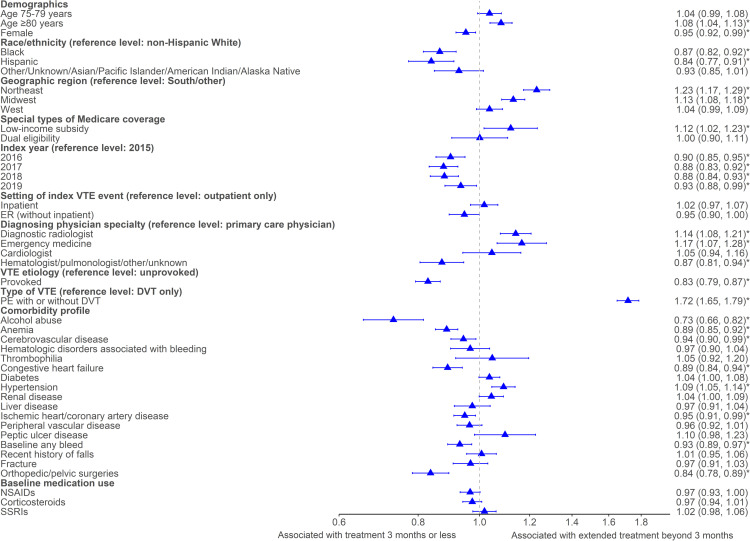
Factors associated with treatment extended beyond 3-months. ^*^P-value < 0.05. A sensitivity analysis was conducted to assess the proportion of OAC-treated patients with extended treatment beyond 6 months and factors associated with extended treatment beyond 6 months (S2 Fig). Among the 66,573 OAC-treated patients who qualified based on having adequate enrollment after the index date, 33,439 (50.2%) had extended treatment for >6 months. Multivariable regression analysis revealed qualitatively similar results as observed among patients with treatment beyond 3 months, with the exception that the strength of association for decreased odds of extended treatment for Hispanic race patients (OR: 0.77 [0.71, 0.84]) compared with non-Hispanic white race, and patients with a Medicare Part D low-income subsidy (OR: 1.23 [1.12, 1.35]) was stronger compared with results from extended treatment beyond 3 months.

Among the OAC-treated cohort, 33.6%, 24.4%, 16.8%, 7.3%, and 18.0%, were on treatment for ≤3 months, >3–≤6 months, >6–≤ 9 months, >9–≤ 12 months, and >12 months, respectively. Extended treatment beyond 3 months was assessed in 73,379 (86.8%) OAC-treated patients with continuous health plan enrollment for >5 months after the index date. Of these patients, 52,833 (72.0%) had extended treatment for >3 months and 20,546 (28.0%) had index treatment for ≤3 months. Notable factors associated with increased odds of extended treatment beyond 3 months included: living in the Northeast region (OR: 1.23 [1.17, 1.29]) compared with the South; having a PE diagnosis (OR: 1.72 [1.65, 1.79]) compared with a DVT only diagnosis; and having a Medicare Part D low-income subsidy (OR: 1.12 [1.02, 1.23]) (Fig 4). Notable factors associated with decreased odds of extended treatment beyond 3 months included: Hispanic race (OR: 0.84 [0.77, 0.91]) compared to non-Hispanic white race; having a provoked VTE (OR: 0.83 [0.79, 0.87]) compared with an unprovoked event; and baseline alcohol abuse (OR: 0.73 [0.66, 0.82]).

## Discussion

This study examined treatment patterns in 169,928 patients with an initial (index) VTE event diagnosed between 2015 and 2019 and observed that 50.3% of patients did not initiate OAC treatment within the 30 days following their VTE event. Among the 49.7% who were treated with an OAC, the majority (74.0%) were treated with DOAC and 26.0% were treated with warfarin, an older drug that requires closer monitoring through regular international normalized ratio measurements, and which is more prone to several drug- and food-based interactions [17].

The percentage of patients with a VTE diagnosis who were untreated is surprisingly high, particularly considering that 29.7% of the untreated patients had a PE diagnosis, which can be life-threatening. While it is conceivable that some VTE diagnoses were based solely on clinical suspicion without confirmatory imaging, this explanation is not likely for the 61.1% of OAC-untreated patients whose first VTE diagnosis was by a radiologist. This indicates a high gap in OAC use in VTE. It is plausible that the ICD codes for VTE are used as a rationale for imaging and not for a confirmatory diagnosis.

In the subgroup that received an OAC, DOAC use increased progressively, and warfarin use decreased steadily over the time period studied. This is consistent with the strong body of evidence that emerged following FDA approval of the DOACs in the 2010s, showing these treatments were at least non-inferior in preventing recurrent VTE, with improved safety due to lower risk of bleeding compared to warfarin [17,18]. This evidence from randomized controlled trials culminated in the recommendations in the 2021 CHEST Guidelines and by ASH in their 2020 Guidelines favoring use of DOACs over vitamin K antagonists (VKA) to treat DVT and PE [8,9]. Throughout the 2010s, the increasing use of DOACs was a driver for increasing overall use of OACs, based on data from both public and commercial health insurance databases [10].

Despite the large-scale national trends showing increased use of DOACs over the past decade, this study indicates that race/ethnicity is a factor associated with underutilization of OACs in general (i.e., comprising both DOACs and VKAs such as warfarin) for VTE. Specifically, Hispanic patients as well as those with race/ethnicity classified as “Other” (such as Asian, Pacific Islander, American Indian, and Alaska Native), had significantly greater odds of being untreated with any OAC in the 30 days following the index event compared to non-Hispanic White patients. More research has been done on use of anticoagulants in atrial fibrillation compared to VTE; however, studies on racial/ethnicity inequities in treatment across the indications consistently highlight the fact that Black patients are less likely to receive any OAC compared to other racial groups [19–21]. The current finding on underutilization of any OAC in Hispanic and “Other” race/ethnicity groups provides valuable additional insights into OAC use among non-Black minorities. Moreover, our observation that eligibility for Medicare part D low-income subsidy poses a risk factor for OAC undertreatment aligns with recent research identifying healthcare and VTE medication costs as common barriers to optimal care following a VTE, even in predominantly insured patient populations.

Previous research has also explored the difference in the type of OAC administered based on race/ethnicity or socioeconomic factors. Underutilization of DOACs versus VKAs for secondary prevention of VTE in Black patients, as well as those with lower household income, was reported in a recent study [22]. In the present study, race/ethnicity, being eligible for Medicare Part-D low-income subsidy, and having dual eligibility with Medicaid did not seem to be a strong predictor of being treated with DOAC versus warfarin.

All DOACs rely on renal elimination in some capacity while warfarin is metabolized in the liver [8,23]. Among the OAC-treated cohort in the present study, patients with renal disease at baseline had lower odds of receiving DOACs than warfarin, which may reflect the consideration of renal function in the choice of OAC. A recent systematic review [24] of the efficacy and safety of anticoagulants in advanced chronic kidney disease reported no significant difference in recurrent VTE events, but reduced bleeding was noted when comparing DOACs to warfarin in patients with a creatinine clearance of 30–50 ml/min based on meta-analysis of data from four randomized controlled trials. The review also indicated a lower risk of recurrent VTE and major bleeding with apixaban compared to warfarin based on observational data in hemodialysis patients. However, additional studies are warranted to further investigate the use of anticoagulation for VTE treatment in individuals with advanced chronic kidney disease, providing robust evidence to guide future prescribing practices [24].

A challenge in clinical management of VTE for patients receiving OACs is deciding whether or not to extend duration of treatment beyond the three months recommended as initial treatment [6,12,14]. A recent study on this question provided evidence for the benefits of OAC treatment beyond an initial three-month course, comparing apixaban, rivaroxaban, and warfarin in patients with an initial VTE event [25]. Significantly lower incidence rates for recurrent VTE hospitalization were observed for apixaban compared with warfarin (HR: 0.69 95% CI: [0.49, 0.99]); however, no significant differences were seen for VTE recurrence between apixaban and rivaroxaban (HR: 0.80 [0.53, 1.19]) nor rivaroxaban and warfarin (HR: 0.87 [0.65, 1.16]), and there were no significant differences in pairwise comparisons for the three treatments in hospitalization for major bleeding. In a separate study with extended treatment of 10 months in apixaban-treated patients and 14 months in warfarin-treated patients, apixaban use compared with warfarin was associated with significantly reduced risks of recurrent VTE (HR: 0.13 [0.03, 0.63]) and major bleeding (HR: 0.56 [0.32, 0.98]) [26]. In the present study, Hispanic race, having a provoked VTE, and baseline alcohol abuse were associated with decreased odds of extended treatment beyond three months in those patients initially treated. Patients with PE were more likely than DVT-only patients to receive OAC treatment beyond three months, as well as beyond six months, attesting to the higher mortality risk and more severe disease burden in this group, compared with DVT-only patients [15,27,28].

Strengths of this study include the large sample size of the Medicare FFS claims database, allowing for real-world analysis of factors associated with timely treatment with OAC versus no timely OAC treatment for VTE. An added advantage of using the Medicare database relates to the use of data from a population with stable coverage for an extended period, for example allowing sensitivity analysis with patients treated beyond six months. This study offers novel findings on treatment rates divided by VTE type (DVT only and PE with or without DVT), by health care setting, and by the specialty of the physician providing the index VTE diagnosis. This study also adds to the growing body of evidence on DOACs which addresses factors associated with VTE patients remaining untreated, despite the better safety of these therapies over warfarin.

Despite these strengths, this study has some limitations which must be considered. This study was subject to the limitations of retrospective studies based on healthcare claims data, such as possible errors or omissions of claims. Comorbidities may also be underestimated due to coding incompleteness and/or inaccuracies. The analyses were intended to assess associations, not to establish causal relationships, and may be subject to uncontrolled confounding. The data used for this analysis may not be generalizable to other populations, for example in patients under 65 years of age or patients with commercial health plans. Since VTE diagnosis was based on ICD codes, under-diagnosis or misdiagnosis may exist, which could impact the observed treatment rates for the overall study population and by VTE type. In addition, VTE diagnosis recorded in the secondary diagnosis position might reflect a history of VTE, and it is unknown how often the VTE ICD codes are used when there is a suspicion of VTE based on clinical symptoms and signs as opposed to when VTE is confirmed. However, requiring two VTE diagnosis claims on two separate dates within 14 days of each other if the first VTE diagnosis was in the ambulatory setting can increase the likelihood of confirming VTE and identifying recent vs. historical events. Identification of OAC prescriptions relied on Part D pharmacy claims, and there is a possibility of misclassifying treated and untreated cohorts, particularly for patients who received OAC or OAC starter packs while transitioning from their inpatient stay. This could contribute to the observed higher odds of remaining untreated when the index VTE event occurred in the inpatient setting, particularly if patients received samples upon discharge and postponed refilling their OAC prescription. Our definition of untreated was based on not having timely treatment, i.e., no observed claim for OAC within 30 days after a VTE diagnosis. Further examination of this group revealed that 23.8% of patients had a fill after 30 days while 76.2% had no fill for OAC throughout follow-up. Lastly, some clinically relevant data such as lab measurements and factors related to physician prescribing behaviors or patient preference were not available in claims data and were not considered in the model.

## Conclusion

In this study of Medicare patients newly diagnosed with VTE, half of the patients were not treated with OAC within the first month from initial diagnosis. Among the untreated, more than one-fourth of patients had a PE diagnosis. Among OAC-treated patients, a higher proportion of patients were treated with DOAC vs. warfarin, with progressively greater use of DOAC in more recent years, consistent with recent guideline recommendations. While various factors might contribute to gap in treatment, factors associated with being untreated such as Hispanic race, Medicare Part-D low-income subsidy, and comorbidity burden might reflect challenges associated with VTE management among disadvantaged populations and in the presence of certain clinical dispositions.

## Supporting information

S1 Table Baseline^1^ characteristics of OAC-treated and untreated patients by VTE type. (DOCX)

S2 Table Multivariable logistic regression assessing factors associated with being untreated^1^ among DVT and PE patients. (DOCX)

S3 Table Baseline^1^ characteristics of patients treated with DOAC and Warfarin. (DOCX)

S1 Fig Factors associated with being untreated with OAC (sensitivity analysis)^#^. *P-value < 0.05. ^*#*^*The sensitivity analysis included medications used to treat key morbidities rather than underlying comorbidities as covariates.*
(TIF)

S2 Fig Factors associated with extended treatment beyond 6 months. *P-value < 0.05.
(TIF)
