# Supplementary material for: Oral anticoagulant use among Medicare patients newly diagnosed with venous thromboembolism (VTE): Factors associated with treatment status
Source: PLoS One. 2025 Apr 17;20(4):e0321106. doi: 10.1371/journal.pone.0321106 (PMC12005561; doi:10.1371/journal.pone.0321106)
Supplement: S2 Table — (DOCX) [file pone.0321106.s002.docx]

| **S2 Table: Multivariable logistic regression assessing factors associated with being untreated^1^ among DVT and PE patients** | | | | | | | | |
| --- | --- | --- | --- | --- | --- | --- | --- | --- |
|  | **Untreated among DVT Patients**  **(N =** **103,843)** | | | | **Untreated among PE Patients^2^**  **(N = 66,085)** | | | |
|  | **Odds Ratio^3^** | **95% CI** | **P-value^4^** | | **Odds Ratio** | **95% CI** | **P-value** | |
| **Demographics at VTE Diagnosis^5^** | | | | | | | | |
| **Age categories (years) (reference: 65-74)** | | | | | | | | |
| 75-79 years | 0.97 | (0.94, 1.01) | 0.136 |  | 0.99 | (0.95, 1.04) | 0.805 |  |
| ≥80 years | 0.98 | (0.95, 1.01) | 0.213 |  | 1.21 | (1.16, 1.26) | <0.001 | * |
| **Sex (reference: male)** | | | | | | | |  |
| Female | 1.18 | (1.15, 1.22) | <0.001 | * | 1.07 | (1.04, 1.11) | <0.001 | * |
| **Race/ethnicity^6^ (reference: non-Hispanic White)** | | | | | | | |  |
| Black | 1.04 | (0.99, 1.09) | 0.101 |  | 1.01 | (0.95, 1.07) | 0.820 |  |
| Hispanic | 1.37 | (1.29, 1.45) | <0.001 | * | 1.36 | (1.25, 1.47) | <0.001 | * |
| Other^7^/Unknown/Asian/Pacific Islander/American Indian/Alaska Native | 1.38 | (1.29, 1.48) | <0.001 | * | 1.15 | (1.05, 1.26) | 0.002 | * |
| **Geographic region (reference: South/other)** | | | | | | | |  |
| Northeast | 1.04 | (1.00, 1.08) | 0.058 |  | 0.84 | (0.80, 0.88) | <0.001 | * |
| Midwest | 0.86 | (0.83, 0.89) | <0.001 | * | 0.82 | (0.78, 0.85) | <0.001 | * |
| West | 0.99 | (0.96, 1.03) | 0.749 |  | 0.83 | (0.79, 0.87) | <0.001 | * |
| **Special types of Medicare coverage at index date** | | | | | | | | |
| Dual Eligibility^8^ | 1.03 | (0.95, 1.11) | 0.508 |  | 1.04 | (0.95, 1.14) | 0.396 |  |
| Low-income subsidy | 1.05 | (0.97, 1.13) | 0.237 |  | 1.27 | (1.16, 1.39) | <0.001 | * |
| **Index year (reference: 2015)** | | | | | | | | |
| 2016 | 0.89 | (0.85, 0.93) | <0.001 | * | 1.50 | (1.41, 1.59) | <0.001 | * |
| 2017 | 0.97 | (0.93, 1.01) | 0.132 |  | 1.58 | (1.49, 1.68) | <0.001 | * |
| 2018 | 0.82 | (0.78, 0.85) | <0.001 | * | 1.33 | (1.26, 1.41) | <0.001 | * |
| 2019 | 0.80 | (0.77, 0.83) | <0.001 | * | 1.36 | (1.28, 1.44) | <0.001 | * |
| **Setting of index VTE event^9^ (reference: outpatient only)** | | | | | | | | |
| Inpatient | 1.48 | (1.43, 1.53) | <0.001 | * | 0.46 | (0.43, 0.50) | <0.001 | * |
| ER (without inpatient) | 0.38 | (0.36, 0.40) | <0.001 | * | 0.33 | (0.30, 0.36) | <0.001 | * |
| **Diagnosing physician specialty^10^ (reference: primary care physician)** | | | | | | | | |
| Diagnostic radiologist | 0.58 | (0.55, 0.60) | <0.001 | * | 0.81 | (0.76, 0.87) | <0.001 | * |
| Emergency medicine | 0.50 | (0.47, 0.54) | <0.001 | * | 0.75 | (0.65, 0.86) | <0.001 | * |
| Hematologist | 0.45 | (0.39, 0.52) | <0.001 | * | 1.04 | (0.82, 1.31) | 0.766 |  |
| Pulmonologist | 1.37 | (1.19, 1.58) | <0.001 | * | 1.50 | (1.32, 1.69) | <0.001 | * |
| Cardiologist | 1.04 | (0.97, 1.12) | 0.285 |  | 1.06 | (0.95, 1.18) | 0.304 |  |
| Other/unknown | 1.28 | (1.21, 1.35) | <0.001 | * | 1.19 | (1.04, 1.37) | 0.012 | * |
| **VTE etiology (reference: unprovoked)** | | | | | | | | |
| Provoked | 0.95 | (0.92, 0.99) | 0.005 | * | 1.15 | (1.10, 1.20) | <0.001 | * |
| **Comorbidity profile in the baseline period** | | | | | | | | |
| Alcohol abuse | 1.38 | (1.27, 1.50) | <0.001 | * | 1.38 | (1.25, 1.52) | <0.001 | * |
| Anemia | 1.19 | (1.15, 1.23) | <0.001 | * | 1.35 | (1.29, 1.40) | <0.001 | * |
| Cerebrovascular disease | 1.36 | (1.31, 1.40) | <0.001 | * | 1.31 | (1.26, 1.37) | <0.001 | * |
| Hematologic disorders associated with bleeding | 1.11 | (1.05, 1.17) | <0.001 | * | 1.19 | (1.11, 1.27) | <0.001 | * |
| Thrombophilia | 0.90 | (0.81, 1.01) | 0.068 |  | 0.93 | (0.82, 1.06) | 0.256 |  |
| Congestive Heart Failure | 1.26 | (1.21, 1.31) | <0.001 | * | 1.68 | (1.61, 1.76) | <0.001 | * |
| Diabetes | 1.11 | (1.08, 1.15) | <0.001 | * | 1.17 | (1.13, 1.22) | <0.001 | * |
| Hypertension | 1.06 | (1.02, 1.10) | 0.004 | * | 1.02 | (0.97, 1.07) | 0.449 |  |
| Renal disease | 0.91 | (0.88, 0.94) | <0.001 | * | 1.04 | (1.00, 1.09) | 0.047 | * |
| Liver disease | 1.09 | (1.04, 1.15) | <0.001 | * | 1.06 | (1.00, 1.13) | 0.060 |  |
| Ischemic heart/coronary artery disease | 1.18 | (1.14, 1.22) | <0.001 | * | 1.17 | (1.13, 1.22) | <0.001 | * |
| Peripheral vascular disease | 1.17 | (1.14, 1.21) | <0.001 | * | 1.29 | (1.23, 1.34) | <0.001 | * |
| Peptic ulcer disease | 1.19 | (1.10, 1.29) | <0.001 | * | 1.15 | (1.04, 1.28) | 0.006 | * |
| Baseline any bleed | 1.22 | (1.18, 1.26) | <0.001 | * | 1.11 | (1.07, 1.16) | <0.001 | * |
| **Recent history of falls** | 1.07 | (1.03, 1.11) | 0.001 | * | 1.28 | (1.22, 1.35) | <0.001 | * |
| **Fracture** | 1.06 | (1.01, 1.11) | 0.017 | * | 1.25 | (1.17, 1.33) | <0.001 | * |
| **Orthopedic/pelvic surgeries** | 0.86 | (0.82, 0.91) | <0.001 | * | 0.97 | (0.91, 1.05) | 0.479 |  |
| **Baseline medication use** | | | | | | | | |
| NSAIDs | 0.95 | (0.93, 0.98) | 0.002 | * | 0.86 | (0.83, 0.89) | <0.001 | * |
| Corticosteroids | 0.84 | (0.81, 0.86) | <0.001 | * | 0.84 | (0.81, 0.87) | <0.001 | * |
| SSRIs | 1.01 | (0.97, 1.04) | 0.708 |  | 1.06 | (1.02, 1.11) | 0.004 | * |

**Abbreviations:**

CI = confidence interval; DVT = deep venous thrombosis; ER = emergency room; NSAID = non-steroidal anti-inflammatory drug; OR = odds ratio; PE = pulmonary embolism; PPI = proton pump inhibitor; SSRI = selective serotonin reuptake inhibitor; VTE = venous thromboembolism.

**Notes:**

1. Untreated is defined as having no claims for apixaban, dabigatran, edoxaban, rivaroxaban, or warfarin during the 30-day period following the index date. Being treated with OACs is defined as having ≥1 claim for apixaban, dabigatran, edoxaban, rivaroxaban, or warfarin during the 30-day period starting from the index date.
2. PE patients with or without DVT.
3. Factors were assessed for their association with being untreated using multivariable logistic regression. An odds ratio over 1 indicates the variable is associated with increased likelihood of being untreated, adjusting for the other factors in the model.
4. An asterisk "*" denotes a two-sided p-value < 0.05.
5. The index date is defined as the start of the study period, January 1, 2014.
6. Race/ethnicity are presented as mutually exclusive categories.
7. U.S. territories such as Puerto Rico and Virgin Island.
8. This category includes patients enrolled in Medicare and getting full Medicaid benefits (i.e., enrolled in Medicaid) and/or assistance with Medicare premiums or cost sharing through the Medicare Savings Program.
9. Patients with claims from multiple settings on their index date were classified as having their index VTE event in the setting that is the first in the following order: inpatient, ER, outpatient. Accordingly, the settings are mutually exclusive.
10. Patients who saw several types of physicians on their index date were classified as having the diagnosing physician specialty with the first specialty in the following order: diagnostic radiologist, emergency medicine, hematologist, pulmonologist, cardiologist, primary care, or other/unknown.
