# Supplementary material for: Oral anticoagulant use among Medicare patients newly diagnosed with venous thromboembolism (VTE): Factors associated with treatment status
Source: PLoS One. 2025 Apr 17;20(4):e0321106. doi: 10.1371/journal.pone.0321106 (PMC12005561; doi:10.1371/journal.pone.0321106)
Supplement: S3 Table — (DOCX) [file pone.0321106.s003.docx]

| **S3 Table: Baseline^1^ characteristics of patients treated with DOAC and Warfarin** | | | | | |
| --- | --- | --- | --- | --- | --- |
|  |  |  |  | **Treated with OAC Cohort**  **(N = 84,538)** | |
|  |  |  |  | **DOAC**  **(N = 62,528)** | **Warfarin**  **(N = 22,010)** |
| **Demographics at index date^2^** | | | | | |
| **Age (years)** | | | | | |
| Mean ± SD | | | | 76.1 ±7.5 | 76.3 ± 7.8 |
| Categories, N (%) | | | | | |
| 65–74 years | | | | 30,547 (48.9%) | 10,680 (48.5%) |
| 75-79 years | | | | 12,958 (20.7%) | 4,406 (20.0%) |
| ≥80 years | | | | 19,023 (30.4%) | 6,924 (31.5%) |
| **Sex, N (%)** | | | | | |
| Female | | | | 39,013 (62.4%) | 14,163 (64.3%) |
| Male | | | | 23,515 (37.6%) | 7,847 (35.7%) |
| **Race/ethnicity,^3^ N (%)** | | | | | |
| American Indian/Alaska Native | | | | 231 (0.4%) | 110 (0.5%) |
| Asian/Pacific Islander | | | | 811 (1.3%) | 236 (1.1%) |
| Black | | | | 5,812 (9.3%) | 2,169 (9.9%) |
| Hispanic | | | | 2,699 (4.3%) | 1,017 (4.6%) |
| Non-Hispanic White | | | | 51,710 (82.7%) | 18,116 (82.3%) |
| Other | | | | 320 (0.5%) | 108 (0.5%) |
| Unknown | | | | 945 (1.5%) | 254 (1.2%) |
| **Geographic region, N (%)** | | | | | |
| Northeast | | | | 11,877 (19.0%) | 3,742 (17.0%) |
| South | | | | 24,182 (38.7%) | 6,497 (29.5%) |
| Midwest | | | | 15,375 (24.6%) | 7,174 (32.6%) |
| West | | | | 10,985 (17.6%) | 4,54 (20.6%) |
| Other^4^ | | | | 109 (0.2%) | 52 (0.2%) |
| **Special types of Medicare coverage at index date, N (%)** | | | | | |
| Dual Eligibility^5^ | | | | 11,790 (18.9%) | 4,406 (20.0%) |
| Low-income subsidy | | | | 13,697 (21.9%) | 5,107 (23.2%) |
| **Index year, N (%)** | | | | | |
| 2015 | | | | 6,996 (11.2%) | 7,399 (33.6%) |
| 2016 | | | | 10,459 (16.7%) | 5,552 (25.2%) |
| 2017 | | | | 12,489 (20.0%) | 4,209 (19.1%) |
| 2018 | | | | 15,753 (25.2%) | 2,819 (12.8%) |
| 2019 | | | | 16,831 (26.9%) | 2,031 (9.2%) |
| **Setting of index VTE event,^6^ N (%)** | | | | | |
| Inpatient | | | | 38,144 (61.0%) | 15,146 (68.8%) |
| Outpatient only | | | | 10,347 (16.5%) | 3,433 (15.6%) |
| ER (without inpatient) | | | | 14,037 (22.4%) | 3,431 (15.6%) |
| **Diagnosing physician specialty,^7^ N (%)** | | | | | |
| Diagnostic radiologist | | | | 47,882 (76.6%) | 16,419 (74.6%) |
| Emergency medicine | | | | 3,398 (5.4%) | 1,041 (4.7%) |
| Hematologist | | | | 431 (0.7%) | 176 (0.8%) |
| Pulmonologist | | | | 742 (1.2%) | 223 (1.0%) |
| Cardiologist | | | | 1,914 (3.1%) | 649 (2.9%) |
| Primary care | | | | 5,541 (8.9%) | 2,518 (11.4%) |
| Other/unknown | | | | 2,620 (4.2%) | 984 (4.5%) |
| **Type of VTE, N (%)** | | | | | |
| DVT only | | | | 32,056 (51.3%) | 11,743 (53.4%) |
| *Acute thrombosis, proximal DVT, lower extremity or abdominal* | | | | 31,716 (50.7%) | 11,626 (52.8%) |
| PE with or without DVT | | | | 30,472 (48.7%) | 10,267 (46.6%) |
| **VTE etiology, N (%)** | | | | | |
| Provoked | | | | 12,545 (20.1%) | 4,972 (22.6%) |
| Unprovoked | | | | 49,983 (79.9%) | 17,038 (77.4%) |
| **Comorbidity profile in the baseline period** | | | | | |
| Charlson Comorbidity Index (CCI) using Quan 2005,^8^ mean ± SD | | | | 2.0 ± 2.0 | 2.2 ± 2.2 |
| **Individual comorbidities, N (%)** | | | | | |
| AIDS | | | | 212 (0.3%) | 102 (0.5%) |
| Alcohol abuse | | | | 1,358 (2.2%) | 508 (2.3%) |
| Anemia | | | | 17,430 (27.9%) | 6,869 (31.2%) |
| Central venous catheter | | | | 2,027 (3.2%) | 1,026 (4.7%) |
| Cerebrovascular disease | | | | 10,522 (16.8%) | 4,075 (18.5%) |
| Hematologic disorders associated with bleeding | | | | 3,471 (5.6%) | 1,364 (6.2%) |
| Ischemic heart/coronary artery disease | | | | 18,581 (29.7%) | 6,909 (31.4%) |
| Dementia | | | | 9,210 (14.7%) | 3,318 (15.1%) |
| Dyspepsia or stomach discomfort | | | | 15,318 (24.5%) | 5,601 (25.4%) |
| Hemiplegia or paraplegia | | | | 1,150 (1.8%) | 473 (2.1%) |
| Hyperlipidemia | | | | 39,451 (63.1%) | 13,480 (61.2%) |
| Obesity | | | | 17,570 (28.1%) | 5,819 (26.4%) |
| Pneumonia | | | | 7,241 (11.6%) | 2,677 (12.2%) |
| Rheumatologic disease | | | | 3,303 (5.3%) | 1,149 (5.2%) |
| Sleep apnea | | | | 8,537 (13.7%) | 3,002 (13.6%) |
| Spinal cord injury | | | | 253 (0.4%) | 91 (0.4%) |
| Thrombophilia | | | | 958 (1.5%) | 380 (1.7%) |
| Varicose veins | | | | 3,199 (5.1%) | 1,033 (4.7%) |
| Congestive heart failure | | | | 7,631 (12.2%) | 3,227 (14.7%) |
| Diabetes | | | | 18,790 (30.1%) | 7,132 (32.4%) |
| Hypertension | | | | 48,590 (77.7%) | 17,275 (78.5%) |
| Renal disease | | | | 11,882 (19.0%) | 5,328 (24.2%) |
| Liver disease | | | | 4,354 (7.0%) | 1,536 (7.0%) |
| COPD | | | | 11,306 (18.1%) | 4,381 (19.9%) |
| Peptic ulcer disease | | | | 1,317 (2.1%) | 605 (2.7%) |
| Inflammatory bowel disease | | | | 987 (1.6%) | 391 (1.8%) |
| Peripheral vascular disease | | | | 10,623 (17.0%) | 4,149 (18.9%) |
| Baseline any bleed | | | | 11,962 (19.1%) | 5,016 (22.8%) |
| **Recent history of falls, N (%)** | | | | 7,767 (12.4%) | 2,788 (12.7%) |
| **Fracture, N (%)** | | | | 5,207 (8.3%) | 2,315 (10.5%) |
| **Orthopedic/pelvic surgeries, N (%)** | | | | 3,811 (6.1%) | 1,691 (7.7%) |
| **HAS-BLED Score, mean ± SD** | | | | 1.5 ± 0.6 | 1.5 ± 0.6 |
| **Baseline medication use, N (%)** | | | | | |
| Antiarrhythmic | | | | 2,870 (4.6%) | 940 (4.3%) |
| Statins | | | | 30,427 (48.7%) | 10,578 (48.1%) |
| Anti-platelets | | | | 4,564 (7.3%) | 1,711 (7.8%) |
| Aromatase inhibitors | | | | 65 (0.1%) | 14 (0.1%) |
| Beta blockers | | | | 20,456 (32.7%) | 7,817 (35.5%) |
| Gastroprotective agents | | | | 21,646 (34.6%) | 8,018 (36.4%) |
| ACE inhibitors | | | | 18,160 (29.0%) | 6,952 (31.6%) |
| ARB | | | | 14,070 (22.5%) | 4,433 (20.1%) |
| NSAIDs | | | | 20,989 (33.6%) | 6,667 (30.3%) |
| Corticosteroids | | | | 35,727 (57.1%) | 12,116 (55.0%) |
| Loop diuretics | | | | 10,522 (16.8%) | 4,413 (20.0%) |
| Potassium-sparing diuretics | | | | 1,964 (3.1%) | 797 (3.6%) |
| Thiazide diuretics | | | | 8,031 (12.8%) | 2,960 (13.4%) |
| Vasodilators | | | | 36,544 (58.4%) | 13,055 (59.3%) |
| PPIs | | | | 18,925 (30.3%) | 7,096 (32.2%) |
| SSRIs | | | | 12,206 (19.5%) | 4,356 (19.8%) |

**Abbreviations:**

ACE = angiotensin-converting enzyme; AIDS = acquired immune deficiency syndrome; ARB = angiotensin receptor blockers; COPD = chronic obstructive pulmonary disease; DVT = deep vein thrombosis; ER = emergency room; HAS-BLED = Hypertension, Abnormal liver/renal function, Stroke history, Bleeding history or predisposition, Labile INR, Elderly, Drug/alcohol usage; LMWH = low molecular weight heparin; NSAID = non-steroidal anti-inflammatory drug; OAC = oral anticoagulant; PE = pulmonary embolism; PPI = proton pump inhibitors; SSRI = selective serotonin reuptake inhibitors; VTE = venous thromboembolism.

**Notes:**

1. The baseline period is defined as the 12 months prior to index date.
2. The index date is defined as the start of the study period, January 1, 2014.
3. Race/ethnicity are presented as mutually exclusive categories.
4. U.S. territories such as Puerto Rico and Virgin Island.
5. This category includes patients enrolled in Medicare and getting full Medicaid benefits (i.e., enrolled in Medicaid) and/or assistance with Medicare premiums or cost sharing through the Medicare Savings Program.
6. Patients with claims from multiple settings on their index date were classified as having their index VTE event in the setting that is the first in the following order: inpatient, ER, outpatient. Accordingly, the settings are mutually exclusive.
7. Patients who saw several types of physicians on their index date were classified as having seen the physician with the first specialty in the following order: diagnostic radiologist, emergency medicine, hematologist, pulmonologist, cardiologist, primary care, or other/unknown.
8. The Charlson Comorbidity Index was defined based on criteria by Charlson (1987), adapted by Deyo (1992), and updated by Quan (2005). Citation: Quan, H. et al., 2005. Coding algorithms and for defining comorbidities in ICD-9-CM and ICD-10 administrative data. Medical Care, 43(11), pp.1130–1139.
