# Supplementary figures and images for: Oral anticoagulant use among Medicare patients newly diagnosed with venous thromboembolism (VTE): Factors associated with treatment status
Source: PLoS One. 2025 Apr 17;20(4):e0321106. doi: 10.1371/journal.pone.0321106 (PMC12005561; doi:10.1371/journal.pone.0321106)

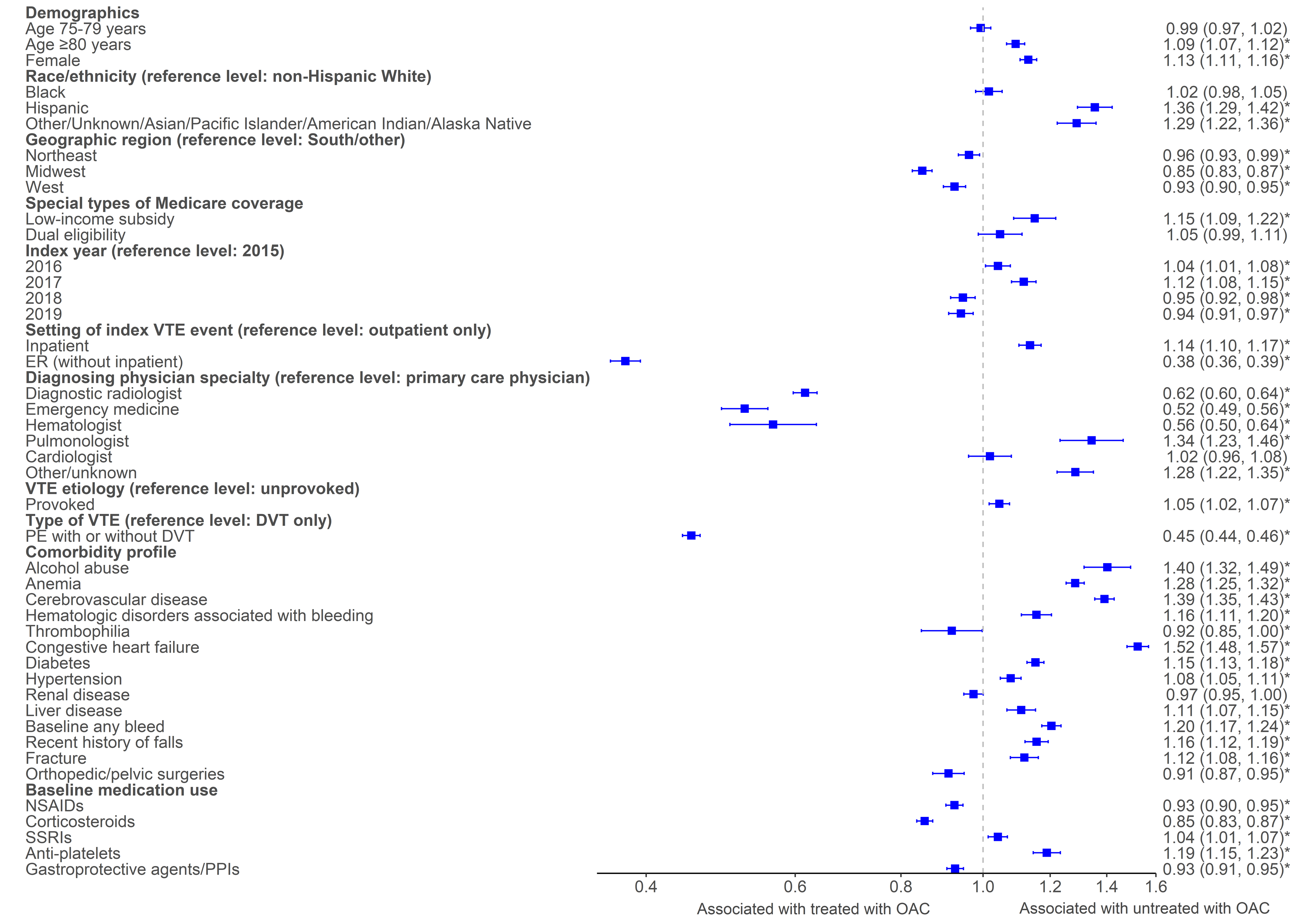

Supplement: S1 Fig — *P-value < 0.05. #The sensitivity analysis included medications used to treat key morbidities rather than underlying comorbidities as covariates. (TIF) [file pone.0321106.s004.tif]

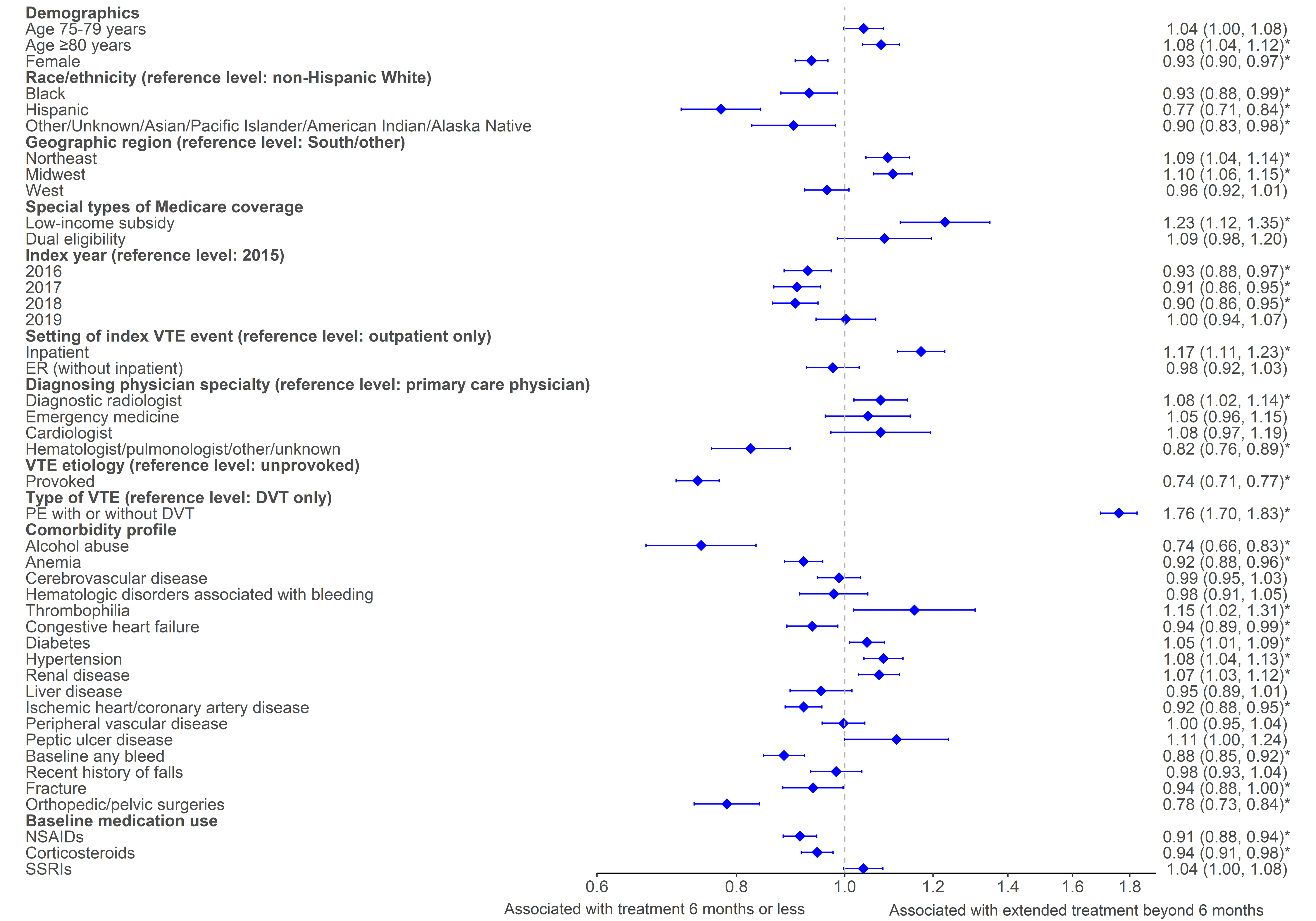

Supplement: S2 Fig — *P-value < 0.05. (TIF) [file pone.0321106.s005.tif]
